# Tadalafil Alone or in Combination with Tamsulosin for the Management for LUTS/BPH and ED

**DOI:** 10.1007/s11934-020-01009-7

**Published:** 2020-10-27

**Authors:** A. Sebastianelli, P. Spatafora, S. Morselli, L. Vignozzi, S. Serni, K. T. McVary, S. Kaplan, S. Gravas, C. Chapple, Mauro Gacci

**Affiliations:** 1 Department of Minimally Invasive and Robotic Urologic Surgery and Kidney Transplantation, Careggi University Hospital, Largo Brambilla 3, 50134 Florence, Italy; 2 Department of Experimental and Clinical Biomedical Science, University of Florence, Florence, Italy; 3 Department of Experimental and Clinical Biomedical Sciences “Mario Serio,” Andrology, Women’s Endocrinology and Gender Incongruence Unit, Careggi Hospital, University of Florence, Florence, Italy; 4 Department of Urology, Stritch School of Medicine, Loyola University Medical Center, Maywood, IL USA; 5 Department of Urology, Icahn School of Medicine at Mount Sinai, New York City, NY USA; 6 Department of Urology, University of Thessaly, Larissa, Greece; 7 Department of Urology, Sheffield Teaching Hospitals, Sheffield, UK

**Keywords:** Benign prostatic hyperplasia, Erectile dysfunction, Combination therapy, Lower urinary tract symptoms, Tadalafil, Tamsulosin

## Abstract

**Purpose of Review:**

Aim of our systematic review is to evaluate and summarize the efficacy and safety of tadalafil alone or in combination with tamsulosin for the management of lower urinary tract symptoms (LUTS)/benign prostatic hyperplasia (BPH) and erectile dysfunction (ED).

**Recent Findings:**

Daily tadalafil, in particular 5 mg, according to retrieved studies, appears to be both safe and effective in treating LUTS/BPH and ED, compared with placebo or tamsulosin. The combination of daily tadalafil 5 mg and tamsulosin 0.4 mg allows a better improvement of LUTS compared with both the monotherapies, even if with an increased, but acceptable and tolerated, adverse events rate. After discontinuation of tamsulosin or tadalafil in patients previously treated with their combination, the improvement of LUTS retains significance compared with baseline.

**Summary:**

Tadalafil 5 mg should be considered a primary treatment option for patients with LUTS/BPH and ED. Evidence highlight an excellent tolerability, safety, and effectiveness profile, both alone or in combination with tamsulosin 0.4 mg. A better efficacy on LUTS relief has been observed for combination therapy, preserving also sexual function. The further switch to monotherapy allows to preserve LUTS relief, but tadalafil only is able to retain ED improvement. Our results support the evidence for a more and more tailored and modular LUTS treatment.

## Introduction

The strong correlation between lower urinary tract symptoms (LUTS) and erectile dysfunction (ED) has now emerged from several preclinical and clinical trials [1]. LUTS due to benign prostatic hyperplasia (BPH) have been recognized as an independent risk factor for ED, thus contributing to the worsening of quality of life (QoL) in the male population [1]. Moreover, medical treatments for LUTS/BPH are able to significantly impact on sexual function. Sexual side effects like ejaculatory dysfunction, reduced or lost libido, and ED have been widely reported in patients treated with alpha blockers (ABs) and 5-alpha reductase inhibitors, the most utilized drugs for the treatment of LUTS/BPH [2, 3].

Phosphodiesterase type 5 inhibitors (PDE5is) represent the gold standard for the treatment of ED. Moreover, PDE5is proved to be effective also for the treatment of LUTS in several preclinical and clinical trials, since they were found to be able to increase oxygenation and blood supply, reduce intraprostatic inflammation, and reduce the smooth muscle tone of the lower urinary tract [4–7]. Indeed, PDE5 was demonstrated to be highly expressed not only in penile corpora cavernosa but also in male bladder, urethra, and prostate [8–10]. Noteworthy, tadalafil 5 mg once daily has been approved and more and more prescribed in the last years as a valuable treatment option for patients complaining LUTS with or without comorbid ED [2, 4–7]. The efficacy of PDE5is on LUTS relief has been evaluated also in combination with ABs. Currently, the only AB approved by the Food and Drug administration for combination treatment with tadalafil is tamsulosin, since a favorable additive effect compared with monotherapy has been proven both on sexual function (increase of International Index of Erectile Function (IIEF) score) and LUTS due to BPH (improvement of International Prostate Symptom Score (IPSS) and maximum flow rate (Qmax)) [11].

Tamsulosin is one of the most prescribed α1-blockers as first line therapy for LUTS associated with benign prostatic enlargement (BPE) or obstruction (BPO) and one of the leading comparators in clinical trials. Therefore, the opportunity to treat both ED and LUTS by using tadalafil alone or in combination with tamsulosin may allow new and more and more tailored therapeutic strategies. However, the balance between efficacy and tolerability represents the crucial point for the treatment of LUTS due to BPH and ED [12].

Aim of our systematic review is to evaluate and summarize the efficacy and safety of tadalafil alone or in combination with tamsulosin for the management of LUTS/BPH and ED.

## Materials and Methods

### Evidence Acquisition

A systematic review of English-language literature was performed up to March 2020 in accordance with the Preferred Reporting Items for Systematic Reviews and Meta-Analyses (PRISMA statement) criteria [13]. Scopus, Medline, PubMed, and Web of Science databases were screened in order to identify clinical trials reporting the use of tadalafil alone or in combination with tamsulosin for the management of patients complaining LUTS/BPH with or without coexisting ED. The following queries were used: “tadalafil” OR “tadalafil combination” OR “tadalafil tamsulosin” AND “lower urinary tract symptoms” OR “LUTS” OR “benign prostatic enlargement” OR “BPE” OR “benign prostatic hyperplasia” OR “BPH.”

Observational, prospective, retrospective, randomized clinical trials and meta-analysis on humans in English language were included. Titles and abstracts were screened, and articles were classified according to treatment.

### Evidence Synthesis

**Fig. 1:**
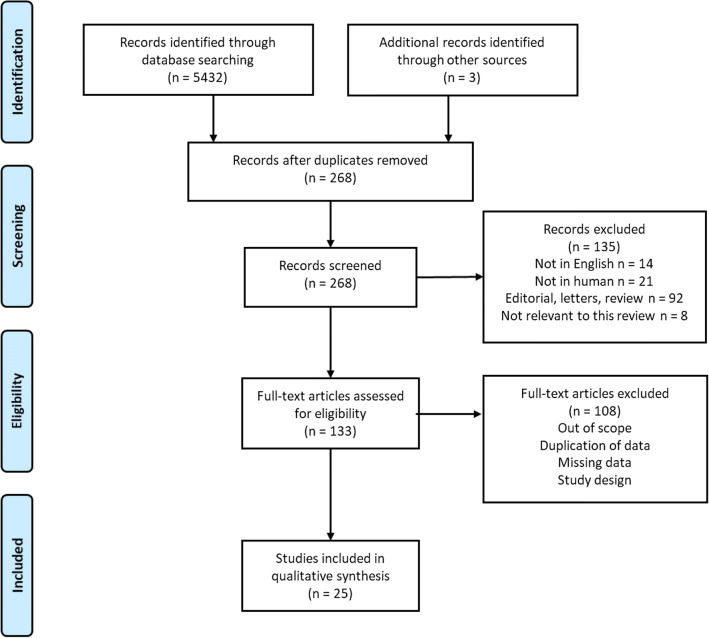
Flow diagram of literature searches according to PRISMA statement

After removing duplicates, a total of 25 papers were identified by the literature search and screened (Fig. 1). A total of 25 articles were included in this review; 9 studies evaluated tadalafil compared with placebo, 7 tadalafil vs tamsulosin, and 9 combination therapy with tadalafil plus tamsulosin vs monotherapy or placebo.

## Results

### Tadalafil vs Placebo

The efficacy and safety outcomes of tadalafil 5 mg vs placebo are shown in Table 1.

**Table 1:** Efficacy and safety outcomes of tadalafil 5 mg vs placebo

Study	No. of patients	Follow-up period	Arms	IPSS	IPSS voiding	IPSS storage	IPSS QoL	IIEF	Qmax (mL/s)	TEAEs
McVary et al. [14] (2007)	281	6 weeks	Tadalafil 5 mg (*N* = 138)	− 2.8	− 1.7	− 1.1	− 0.5	6.0	1.1	3.6%
			Placebo (*N* = 143)	− 1.2	− 0.8	0.4	− 0.2	0.6	1	1.4%
			*p* value	0.003	0.01	0.003	0.017	< 0.001	0.46	
Roehrborn et al. [15] (2008)	1058	12 weeks	Tadalafil 5 mg (*N* = 212)	− 4.9	− 2.9	− 2	− 0.9	7	1.6	30.7%
			Placebo (*N* = 211)	− 2.3	− 1.3	− 1	− 0.5	2.2	1.2	21.2%
			*p* value	< 0.001	< 0.001	< 0.01	< 0.01	< 0.001	> 0.05	
Porst et al. [16] (2009)	581	12 weeks	Tadalafil 5 mg (*N* = 117)	− 4.2				6.8	1.7	25.6%
			Placebo (*N* = 115)	− 2.1				2	1.9	20.9%
			*p* value	0.004				< 0.001	0.75	
Porst et al. [17] (2011)	325	12 weeks	Tadalafil 5 mg (*N* = 161)	− 5.6	− 3.3	− 2.3	− 1	6.7	1.6	26.1%
			Placebo (*N* = 164)	− 3.6	− 2.3	− 1.3	− 0.7	2	1.1	22%
			*p* value	0.004	0.02	0.002	0.013	< 0.001	0.30	
Egerdie et al. [18] (2012)	606	12 weeks	Tadalafil 5 mg (*N* = 208)	− 6.1	− 3.6	− 2.5	− 1	6.5	1.6	27.4%
			Placebo (*N* = 200)	− 3.8	− 2.2	− 1.6	− 0.8	1.8	1.2	19.5%
			*p* value	< 0.001	< 0.001	< 0.001	0.08	< 0.001	0.19	
Takeda et al. [19] (2012)	422	12 weeks	Tadalafil 5 mg (*N* = 140)	− 5	− 3.3	− 1.6	− 0.7		0.6	40.7%
			Placebo (*N* = 140)	− 3.7	− 2.4	− 1.4	− 0.4		1.4	37.9%
			*p* value	0.035	0.033	0.487	0.022		0.094	
Takeda et al. [20] (2014)	610	12 weeks	Tadalafil 5 mg (*N* = 306)	− 6	− 4	− 2	− 1.1		1.20	28.4%
			Placebo (*N* = 304)	− 4.5	− 3.1	− 1.4	− 0.9		0.60	25%
			*p* value	< 0.001	0.002	0.002	0.038		0.095	
Matsukawa et al. [21] (2019)	105	52 weeks	Tadalafil 5 mg (*N* = 94)	− 6.9	− 4.2	− 2.3	− 1.9		2.9	

*IPSS*, International Prostate Symptom Score; *QoL*, quality of life; *IIEF*, International Index of Erectile Function; *Qmax*, peak urine flow rate; *TEAE*, treatment-emergent adverse events; *N*, number of participants randomized; *p value*, difference compared to placebo

In 2007, McVary et al. published the results of the first randomized clinical trial (RCT) on the use of tadalafil to treat LUTS due to BPH [14]. A total of 281 men with a history of LUTS due to BPH for at least 6 months were randomly assigned (1:1) to take placebo for 12 weeks or tadalafil 5 mg daily for 6 weeks and a dose escalation to 20 mg for the subsequent 6 weeks. LUTS, evaluated by International Prostate Symptom Score (IPSS), were significantly improved in tadalafil arm at 6 (− 2.8 vs − 1.2 IPSS points) and 12 weeks (− 3.8 vs − 1.7). Both storage and voiding IPSS subscores were significantly improved too. However, uroflowmetry parameters, at the end of the trial, were similar in the 2 groups. As expected, tadalafil improved also the International Index of Erectile Function (IIEF-EF) domain in the 156 men sexually active, complaining ED. Moreover, a low incidence of treatment-emergent adverse events (TEAEs) was reported, since the incidence of dyspepsia, back pain, and headache, the most common TEAEs, was < 4.3% and the discontinuation due to TEAEs was 3.6% compared with 1.6% in the placebo arm.

A dose-finding study was published in 2008 [15]. Roehrborn et al., in a 12-week RCT including 1058 men, compared with placebo 4 different daily dosage of tadalafil: 2.5 mg vs 5 mg vs 10 mg vs 20 mg. LUTS significantly improved in all the tadalafil groups compared with placebo, in a clinically meaningful manner. Indeed, IPSS decrease was − 3.9 for 2.5 mg, − 4.9 for 5 mg, − 5.2 for 10 mg, and − 5.2 for 20 mg, compared to − 2.3 of placebo. Interestingly, the IPSS least squares mean change in tadalafil 5 mg group was − 2.6 points, similarly to those reported in tamsulosin trials [22]. Moreover, all doses of tadalafil were well tolerated and few patients discontinued the study, leading to the conclusion that daily tadalafil 5 mg was effective in improving LUTS due to BPH with a certain risk–benefit profile.

A post hoc analysis on 581 men by Porst et al. confirmed that all tadalafil doses allowed a significant IPSS improvement versus placebo (all *p* values < 0.05). Moreover, in this population of sexually active men with moderate to severe LUTS and ED, daily 5-mg tadalafil offered the best risk–benefit profile, both for urinary and sexual function [16].

Dmochowski et al. confirmed in 2010 the significant effect of tadalafil 20 mg on IPSS, compared with placebo (mean difference between treatments: − 4.2, *p* < 0.001), but they failed to prove a significant impact of tadalafil on urodynamic parameters. Indeed, no statistically or clinically significant differences were observed between tadalafil and placebo groups in maximum urinary flow rate (Qmax), detrusor pressure at Qmax (PdetQmax), or bladder capacity [23].

In 2011, always Porst et al. compared tadalafil 5 mg with placebo in a RCT, evaluating 325 men with LUTS for at least 6 months, IPSS ≥ 13, and maximum urine flow rate (Qmax) ≥ 4 and ≤ 15 mL/s. Tadalafil was able to significantly improve total IPSS compared with placebo (− 1.9; *p* = 0.004); also, voiding (*p* = 0.02), storage IPSS subscores (*p* = 0.002), QoL index (*p* = 0.013), frequency (question 2 of IPSS; *p* < 0.001), and urgency (question 4; *p* = 0.035) were significantly improved in the tadalafil group. Moreover, a significant improvement of Patient Global Impression (PGI-I) (better: 74.2% vs 57.6%; *p* = 0.003) and Clinician Global Impression (CGI-I) (better: 71.0% vs 55.1%; *p* = 0.009) was observed in the tadalafil group. In sexually active men with both LUTS/BPH and ED, erectile function significantly improved when compared with placebo (IIEF: + 6.7 vs + 2; *p* < 0.001). The most common AEs were headache and back pain [17].

A multinational phase 3 RCT was conducted by Egerdie et al. in 2012 in order to assess the impact of once daily tadalafil 2.5 or 5 mg on men with ED standing for at least 3 months and LUTS/BPH. IIEF-EF domain scores improved in both the tadalafil 2.5 mg group (*n* = 198) and the tadalafil 5 mg group (*n* = 208), compared with placebo (*n* = 200). However, only tadalafil 5 mg, but not 2.5 mg, was able to significantly improve total IPSS; the least squares mean change from baseline was − 4.6 in tadalafil 2.5 mg arm, − 6.1 in tadalafil 5 mg arm, and − 3.8 in placebo arm (*p* < 0.001). Moreover, tadalafil 5 mg significantly improved Sexual Encounter Profile Question 3 (SEP Q3) and BPH Impact Index (BII) (*p* < 0.001) [18].

Always in 2012, Takeda et al. published a 12-week placebo-controlled dose-finding RCT with a 42-week open-label extension. The authors compared tadalafil 2.5 mg (*n* = 142) or tadalafil 5 mg (*n* = 140) with placebo (*n* = 140) in Japanese men ≥ 45 years with moderate to severe LUTS due to BPH for 12 weeks, followed by an open-label extension (OLE) with tadalafil 5 mg (*n* = 394) for 42 weeks. Only tadalafil 5 mg showed a significant least squares (LS) mean difference in total IPSS compared with placebo (∆ = − 1.2; *p* = 0.035). Moreover, both obstructive (*p* = 0.033) and IPSS QoL subscores (*p* = 0.022) were significantly improved and persisted over the OLE phase [19]. These findings were confirmed in 2014 by the same group, comparing tadalafil 5 mg (*n* = 306) with placebo (*n* = 304) in Japanese and Korean men with LUTS/BPH. Total (− 1.5 ± 0.5 (− 2.4 to − 0.6); *p* < 0.001), storage (− 0.6 ± 0.2 (− 0.9 to − 0.2); *p* = 0.002), voiding (− 0.9 ± 0.3 (− 1.5 to − 0.3); *p* = 0.002), and QoL IPSS (− 0.2 ± 0.1 (− 0.4 to − 0.0); *p* = 0.038) were all significantly improved compared with placebo. Nasopharyngitis (tadalafil vs placebo: 4.2 vs 3.3%), dyspepsia (3.9 vs 0.7%), and headache (2.9 vs 2%) were the most common TEAEs [20].

Recently, Matsukawa et al. reported 1-year outcomes from a prospective urodynamic study. The analysis included 94 men with LUTS/BPH treated with daily tadalafil 5 mg for 12 months. At 3-month follow-up, there was significant improvement from baseline of total IPSS (− 5.4; *p* < 0.001), voiding (− 3.1; *p* < 0.001), storage (− 2.3; *p* < 0.001), QoL (− 1.6; *p* < 0.001), overactive bladder symptom score (OABSS) (− 1.7; *p* < 0.001), and benign prostatic hyperplasia impact index (BII) (− 2.7; *p* < 0.001). Interestingly, the improvement was even stronger at 12 months. Moreover, Qmax significantly increased by 2.9 mL/s at 12-month follow-up (*p* < 0.001), and detrusor overactivity, baseline diagnosed at cystometry in 49 men, was no more detected after 3 months of treatment in 15 patients (30.6%; *p* = 0.02), and in 22 at the end of the trial (44.9%, *p* < 0.001). Mean bladder outlet obstruction index, 59.5 at baseline, significantly and progressively decreased until 42.9 (*p* < 0.001) after 12 months [21].

### Tadalafil vs Tamsulosin

The efficacy and safety outcomes of tadalafil 5 mg vs tamsulosin are shown in Table 2.

**Table 2:** Efficacy and safety outcomes of tadalafil 5 mg vs tamsulosin

Study	No. of patients	Follow-up period	Arms	IPSS	IPSS voiding	IPSS storage	IPSS QoL	IIEF	Qmax (mL/s)	TEAEs
Kim et al. [24] (2011)	151	12 weeks	Placebo (*N* = 51)	− 4.2	− 2.7	− 1.5	− 0.9		2.3	3.9%
			Tadalafil 5 mg (*N* = 51)	− 5.8	− 3.7	− 2.1	− 1.2		2.5	13.7%
			*p* value	0.07	0.10	0.15	0.21		0.84	
			Tamsulosin 0.2 mg (*N* = 49)	− 5.4	− 3.6	− 1.8	− 1.0		2.1	26.5%
			*p* value	0.19	0.15	0.52	0.59		0.83	
Yokoyama et al. [25] (2013)	612	12 weeks	Placebo (*N* = 154)	− 3	− 1.9	− 1.1	− 0.5		2.1	19.5%
			Tadalafil 5 mg (*N* = 155)	− 5.1	− 3	− 1.7	− 0.8		1.3	30.3%
			*p* value	0.001	0.005	0.021	0.013		0.14	
			Tamsulosin 0.2 mg (*N* = 152)	− 5.6	− 3.8	− 1.7	− 1.1		2.1	24.3%
Oelke et al. [26••] (2012)	511	12 weeks	Placebo (*N* = 172)	− 4.2	− 2.6	− 1.6	− 1.0		1.2	20.3%
			Tadalafil 5 mg (*N* = 171)	− 6.3	− 4.1	− 2.2	− 1.3	4	2.4	23.4%
			*p* value	0.001	< 0.001	0.055	0.022	< 0.001	0.009	
			Tamsulosin 0.4 mg (*N* = 168)	− 5.7	− 3.5	− 2.2	− 1.1	− 0.4	2.2	23.8%
			*p* value	0.023	0.026	0.055	0.546	0.699	0.014	
Giuliano et al. [4, 27] (2013)	511	12 weeks	Placebo (*N* = 105)					2.1		
			Tadalafil 5 mg (*N* = 106)					6		
			*p* value					< 0.001		
			Tamsulosin 0.4 mg (*N* = 99)					1.7		
			*p* value					0.699		
Zhang et al. [28] (2019)	909	12 weeks	Placebo (*N* = 361)	− 4.08				1.88	1.5	17.2%
			Tadalafil 5 mg (*N* = 363)	− 5.49				5.24	1.9	18%
			*p* value	< 0.001	< 0.001	0.077	0.220	< 0.001	0.568	
			Tamsulosin 0.2 mg (*N* = 185)	− 4.92				2.64	2	11.9%
			*p* value	0.105	0.061	0.334	0.216	> 0.05	0.246	
Pogula et al. [29] (2019)	100	12 weeks	Tadalafil 5 mg (*N* = 50)	− 0.62			− 0.26		2.4	
			Tamsulosin 0.4 mg (*N* = 50)	− 2.76			− 0.76		4	
			*p* value*	0.438			0.127		0.002	

*IPSS*, International Prostate Symptom Score; *QoL*, quality of life; *IIEF*, International Index of Erectile Function; *Qmax*, peak urine flow rate; *TEAE*, treatment-emergent adverse events; *N*, number of participants randomized; *p value*, difference compared to placebo; p value*, difference compared to tamsulosin

In 2011, Kim et al. enrolled 151 Korean men, randomly assigned to receive once daily tadalafil 5 mg, tamsulosin 0.2 mg, or placebo for 12 weeks. At endpoint, the authors observed only a numerical but not statistically significant improvement of total IPSS in both tadalafil and tamsulosin arms, compared with placebo (− 5.8 vs − 5.4 vs − 4.2; *p* < 0.05). The same findings were reported for the other efficacy outcomes evaluated, concluding that larger studies in Asian men were needed [24].

The following year, Yokoyama et al. published the results of their 12-week RCT evaluating 151 Asian men complaining LUTS treated with tadalafil 2.5 mg, 155 with tadalafil 5 mg, 152 with tamsulosin 0.2 mg as active control, and 154 with placebo. Total IPSS decrease was − 5.0 ± 0.4 in the tadalafil 2.5 mg group, − 5.1 ± 0.4 in tadalafil 5 mg, − 5.6 ± 0.4 in tamsulosin 0.2 mg, and − 3.0 ± 0.4 in the placebo group. Moreover, tadalafil 5 mg (− 1.7, *p* = 0.021), but not tadalafil 2.5 mg (− 1.5; *p* = 0.072), significantly improved IPSS storage subscore compared with placebo, and a similar reduction was observed with tamsulosin 0.2 mg (− 1.7 ± 0.2) [25].

The severity of TEAEs was mild to moderate in 96.9% of subjects, being the most common: myalgia, headache, back pain, nasopharyngitis, and dizziness. In the placebo group, 19.5% of men experienced ≥ 1 TEAE, compared with 29.8% in tadalafil 2.5 mg, 30.3% in tadalafil 5.0 mg, and 24.3% the in tamsulosin group.

Always in 2012, Oelke et al. compared, in a 12-week RCT, 171 men in the tadalafil 5 mg group, 168 in the tamsulosin 0.4 mg group, and 172 in the placebo group, after 4 weeks of placebo run-in. Compared with placebo, total IPSS significantly improved in both tadalafil (− 6.3 from baseline; ∆placebo = − 2.1; *p* = 0.001) and tamsulosin arms (− 5.7 from baseline; ∆placebo = − 1.5; *p* = 0.023). Interestingly, also a significant improvement of Qmax was observed in both tadalafil (2.4 mL/s; *p* = 0.009) and tamsulosin (2.2 mL/s; *p* = 0.014) groups. Nevertheless, tadalafil, but not tamsulosin, was able to significantly improve IPSS QoL Index and Treatment Satisfaction Scale–BPH compared with placebo (both *p* < 0.05 vs both *p* > 0.1). Likewise, IIEF-EF improved vs placebo with tadalafil (+ 4.0; *p* < 0.001) but not with tamsulosin (− 0.4; *p* = 0.699). In the placebo group, 20.3% of men experienced ≥ 1 TEAE, compared with 23.4% in tadalafil and 23.8% in the tamsulosin group. Headache, nasopharyngitis, back pain, dizziness, and dyspepsia were the most common adverse events, and the TEAEs discontinuation rate was similar in the 3 groups (1.2% vs 1.2% vs 0.6%). Despite the interesting results, the authors stated that the study was not powered for a direct comparison of tadalafil and tamsulosin [26••].

Daily tadalafil 5 mg improved EF, but also sexual satisfaction and ejaculatory function, in men with LUTS/BPH and ED, as shown in an RCT by Giuliano et al., compared with placebo and tamsulosin 0.4 mg, which was associated with a decrease of ejaculatory and orgasmic frequency and overall satisfaction [27].

In 2014, Oelke et al. published the results of an RCT aimed to evaluate treatment satisfaction with daily tadalafil 5 mg or tamsulosin 0.4 mg compared with placebo in men with LUTS/BPH. A total of 171 men received tadalafil 5 mg, 168 tamsulosin 0.4 mg, and 171 placebo for 12 weeks. Men treated with tadalafil showed an overall satisfaction statistically significant superior to placebo (*p* = 0.005); conversely, men treated with tamsulosin or placebo showed similar results. Also, the “satisfaction with efficacy” domain was significantly greater with tadalafil (*p* = 0.003) but not with tamsulosin compared with placebo. Tadalafil was evaluated as “effective/very effective” by 66.5% of men (*p* = 0.011 vs placebo), 71.8% were generally “very satisfied/satisfied with their medication” (*p* < 0.003), and 65.0% would “definitely/probably continue therapy” (*p* = 0.035) [30].

A phase 3, randomized, double-blind, parallel, placebo- and tamsulosin-controlled study was carried out by Zhang et al. in 2018. A total of 909 Asian men were randomized in a 2:2:1 proportion to receive for 12 weeks tadalafil 5 mg (*n* = 363), placebo (*n* = 361), or tamsulosin 0.2 mg (*n* = 185). Men treated with tadalafil 5 mg experienced a 5.49 IPSS reduction, significantly better than placebo (∆placebo = − 1.41; *p* < 0.001); conversely, men in the tamsulosin arm showed similar results when compared with placebo (− 4.9 vs − 4.08; *p* = 0.105). Also, PGI-I and CGI-I were significantly better in the tadalafil group. Qmax in tadalafil and tamsulosin arm was numerically, but not statistically, significantly improved vs placebo (+ 1.9 mL/s vs + 2.0 mL/s vs + 1.5 mL/s; *p* = 0.568 and *p* = 0.246, respectively). As expected, IIEF-EF improvement was significant in the tadalafil group (∆ = 5.24; *p* < 0.001) but not in the tamsulosin group (∆ = 2.64; *p* > 0.05) vs placebo (∆ = 1.88) [28].

Pogula et al. compared tadalafil 5 mg with tamsulosin 0.4 mg in a 12-week RCT, enrolling 50 men with LUTS/BPH in each group. Total IPSS score was improved from the baseline in both groups, without a statistically significant difference between the 2 treatment arms (tadalafil vs tamsulosin: − 0.62 vs − 2.76; *p* = 0.438. Qmax was improved in both groups too, compared with baseline; nevertheless, men in the tamsulosin group experienced a statistically significant improvement compared with tadalafil (tadalafil vs tamsulosin: + 2.4 vs + 4; *p* = 0.002). Only tadalafil showed a significant improvement of ED [29].

### Combination vs Monotherapy

The efficacy and safety outcomes of tadalafil alone or in combination with tamsulosin are shown in Table 3.

**Table 3:** Efficacy and safety outcomes of tadalafil alone or in combination with tamsulosin

Study	No. of patients	Follow-up period	Arms	IPSS	IPSS voiding	IPSS storage	IPSS QoL	IIEF	Qmax (mL/s)	TEAEs
Bechara et al. [31] (2008)	30	6 weeks	Tadalafil 20 mg + tamsulosin 0.4 mg (*N* = 15)	− 9.2			− 2.5	8.2	3	55.5%
			Tamsulosin 0.4 mg (N = 15)	− 6.7			− 1.8	1.9	2.1	18.5%
			*p* value	< 0.05			< 0.05	< 0.001	< 0.05	
Regadas et al. [32] (2013)	40	4 weeks	Tadalafil 5 mg + tamsulosin 0.4 mg (*N* = 20)	− 9.75	− 6.3	− 3.9			1	
			Tamsulosin 0.4 mg (*N* = 20)	− 6	− 4.5	− 1.5			1.4	
			*p* value	0.01	0.01	0.05			0.65	
Singh et al. [33] (2014)	133	12 weeks	Tadalafil 10 mg + tamsulosin 0.4 mg (*N* = 44)	− 11.73			− 4.5	6.39	3.66	
			Tadalafil 10 mg (*N* = 44)	− 6.83			− 4.04	5.5	2.63	
			Tamsulosin 0.4 mg (*N* = 45)	− 10.67			− 4.11	4.4	3.11	
Karami et al. [34] (2016)	183	12 weeks	Tadalafil 20 mg + tamsulosin 0.4 mg (*N* = 61)	− 11.1	− 8	− 3.3		7.6	3.5	31.03%
			Tadalafil 20 mg (*N* = 61)	− 8.6	− 7.1	− 2.1		7.8	1.5	23.3%
			Tamsulosin 0.4 mg (*N* = 61)	− 10.1	− 7.1	− 2.9		4.6	3.3	11.8%
Kim et al. [35] (2017)	510	12 weeks	Tadalafil 5 mg + tamsulosin 0.2 mg (*N* = 20)							
			Tadalafil 5 mg + tamsulosin 0.4 mg (*N* = 20)	− 9.46	− 6.56	− 2.91	− 1.48	9.17	6.64	20.25%
			Tadalafil 5 mg (*N* = 20)	− 8.14	− 5.56	− 2.57	− 1.34	9.49	5.68	14.04%
			*p* value	0.032	0.014	0.189	0.269	0.588	0.24	
Sebastianelli et al. [34] (2019)	75	12 weeks	Tadalafil 5 mg + tamsulosin 0.4 mg (*N* = 50)	− 7	− 3.5	− 3	− 1.8	5.7	4.2	22%
			Tadalafil 5 mg (*N* = 25)	− 5.2	− 2	− 3.1	− 1.3	6.1	2.2	16%
			*p* value	0.084	0.006	0.08	0.321	0.255	0.027	

*IPSS*, International Prostate Symptom Score; *QoL*, quality of life; *IIEF*, International Index of Erectile Function; *Qmax*, peak urine flow rate; *TEAE*, treatment-emergent adverse events; *N*, number of participants randomized; *p value*, difference compared to tamsulosin; p value*, difference compared to tadalafil

A pilot crossover RCT aimed to assess the efficacy of tamsulosin plus tadalafil vs tamsulosin in the treatment of men with LUTS/BPH was published by Bechara et al. in 2008. For 45 days, 15 men received daily tamsulosin 0.4 mg plus tadalafil 20 mg and 15 men tamsulosin 0.4 mg plus placebo, then treatments were switched and lasted for further 45 days. After 6 weeks of treatment, total IPSS was significantly improved from baseline in both groups; however, patients treated with combination therapy showed a more remarkable significant improvement compared with tamsulosin alone (combination vs tamsulosin: − 9.2 vs − 6.7; *p* < 0.05). The same findings were reported for IPSS QoL (combination vs tamsulosin: − 2.5 vs − 1.8; *p* < 0.05) and visual analogical scale (VAS) (combination vs tamsulosin: − 3.7 vs − 2.3; *p* < 0.05). Qmax and PVR significantly changed from baseline, without any statistically significant difference in the 2 groups (combination vs tamsulosin: + 3 vs + 2.1; *p* > 0.05; and − 38.7 vs − 35.2; *p* > 0.05). IIEF significantly improved only in the combination arm. At the end of the trials, all patients preferred the combination treatment period. Nevertheless, TEAEs were reported by 55.5% of men in the combination arm and by 18.5% in the tamsulosin arm. The most frequently reported TEAs were headache, hypotension (not clinically significant), and dyspepsia, all more common in the combination group [31].

Urodynamic effects of combination of daily tamsulosin 0.4 mg plus tadalafil 5 mg vs tamsulosin 0.4 mg plus placebo were analyzed by Regadas et al. in a 4-week RCT involving 40 men. Qmax similarly increased in both groups (combination vs tamsulosin: + 1 vs + 1.4; *p* = 0.65). Conversely, PdetQmax and bladder outlet obstruction index (BOOI) significantly improved only in the combination arm (− 13 vs − 1.1; *p* = 0.03; − 116.8 vs − 4.3; *p* = 0.02). At the end of the trial, total, voiding, and storage IPSS were significantly better in the combination arm compared with tamsulosin (− 9.75 vs – 6, *p* = 0.01; − 6.3 vs − 4.5, *p* = 0.01; − 3.9 vs − 1.5, *p* = 0.05) [32].

Always in 2014, Singh et al. enrolled 133 Indian men with LUTS/BPH in order to carry out a 12-week RCT, aiming to compare 45 patients treated with tamsulosin 0.4 mg, 44 patients daily tadalafil 10 mg/day, and 44 combination of the 2 drugs. Total IPSS significantly improved in all the three groups (− 50.90%, *p* < 0.05 vs − 33.50%, *p* < 0.05 vs − 53.90%, *p* < 0.05, respectively). Interestingly, the same trend was reported for Qmax and IIEF5 score (Qmax: + 33.99%; *p* < 0.05 vs + 29.78%; *p* < 0.05 vs + 37.04%; *p* < 0.05; IPSS: + 39.28%; *p* < 0.05 vs + 45.96%; *p* < 0.05 vs + 60.23%, *p* < 0.05, respectively). However, the improvement was better with combination treatment compared with monotherapy of both drugs. All treatments were generally well tolerated, and the most frequent reported TEAEs were dyspepsia, heartburn, headache, flushing, myalgia, and backache [33].

An RCT from Iran was published in 2016 enrolling 61 patients with LUTS/BPH and ED for each arm, randomly selected to receive daily tadalafil 20 mg or daily tamsulosin 0.4 mg or combination of the 2 drugs for 12 weeks. Total, storage, and voiding IPSS were significantly improved compared to baseline in all the 3 arms, being significantly better in the combination group (all *p* < 0.05). Qmax significantly improved from baseline only in the 2 tamsulosin arms. Otherwise, IIEF was significantly improved from baseline only in the 2 tadalafil arms. Myalgia, headache, backpain, nasopharyngitis, and dizziness were the most common TEAEs reported. Moreover, 3 men in the combination group (5%) discontinued the trial due to TEAEs vs 1 (1.6%) in tadalafil and 2 (3.3%) in the tamsulosin arm [34].

In 2017, Kim et al. carried out an RCT on 510 men with LUTS/BPH and ED. Subjects were randomly selected to be treated for 12 weeks with daily tamsulosin 0.4 mg plus tadalafil 5 mg or tamsulosin 0.2 mg plus tadalafil 5 mg or tadalafil 5 mg plus placebo. A 12-week extension period was then carried out with tamsulosin 0.4 mg plus tadalafil 5 mg involving 440 men still available to continue the trial. Total, voiding, and storage IPSS significantly improved from baseline in all the 3 treatment arms. A statistically significant difference between combination of tamsulosin 0.4 mg plus tadalafil 5 mg and tadalafil 5 mg plus placebo was observed for total and voiding IPSS (− 9.46 vs − 7.81, *p* = 0.032; − 6.56 vs − 5.39, *p* = 0.01); storage IPSS similarly improved (− 2.9 vs − 2.4, *p* = 0.18). Combination of tamsulosin 0.2 mg plus tadalafil 5 mg and tadalafil 5 mg alone reached similar total, voiding, and storage IPSS improvement. Qmax similarly improved in the 3 treatment arms (+ 6.64 mL/s vs + 4.88 mL/s vs + 5.73 mL/s, *p* = 0.2). An IIEF score ≥ 26 was reported by 46.7% in the combination group with tamsulosin 0.4 mg, 50.68% in combination with tamsulosin 0.2 mg, and 46.71% in tadalafil monotherapy. Mean total IPSS change in the extension period was − 0.97, − 1.28, and − 2.45, for patients previously treated with combination tamsulosin 0.4 mg, combination tamsulosin 0.2 mg, and tadalafil monotherapy, respectively. The overall occurrence of TEAEs was 14.11% vs 8.43% vs 5.85%. Men treated with tamsulosin 0.4 mg combination had a significantly higher TEAEs rate (*p* < 0.05) compared with tadalafil alone. However, 11.59% of men reported at least one TEAE in the extension period. TEAEs were mild to moderate and generally resolved without sequelae. Headache, nasal congestion, and ocular hyperemia were the most frequent TEAEs [35].

A meta-analysis of 4 RCT comparing combination therapy of tamsulosin 0.4 mg and tadalafil vs tadalafil alone was published in 2019 by Zhou et al. Of the 4 retrieved trials, two evaluated daily tadalafil 5 mg (one not in English language), one tadalafil 10 mg, and one tadalafil 20 mg. Despite the limitations of this study, the authors confirmed that, compared with tadalafil alone, combination therapy was associated with a significant improvement of total IPSS, QoL, and Qmax, with a more remarkable improvement of voiding IPSS. However, a higher rate of TEAEs and a higher discontinuation rate were recorded for combination therapy [36].

Combination therapy with tadalafil 5 mg and tamsulosin 0.4 mg (*n* = 50) was compared with tadalafil 5 mg monotherapy (*n* = 25) by Sebastianelli el al. in 2019 compared to baseline; after 12 weeks, total (combination vs tadalafil: − 7 vs − 5.2, *p* = 0.08), voiding, storage (− 3 vs − 3.1, *p* = 0.08), and QoL (− 1.8 vs − 1.3, *p* = 0.3) IPSS significantly improved in both groups. However, along with Qmax (+ 4.2 vs + 2.2, *p* = 0.027), voiding IPSS (− 3.5 vs − 2; *p* = 0.006) was significantly better in the combination arm. IIEF improvement was similar between the two treatment arms (+ 5.7 vs + 6.1, *p* = 0.255). The incidence of TEAEs was numerically but not significantly higher in the combination group (22% vs 16%, *p* = 0.07). No one experienced severe TEAEs. Headache, nasopharyngitis, back pain, and dizziness were the most frequent TEAEs [37••].

Always in 2019, the same group evaluated the impact of tadalafil 5 mg or tamsulosin 0.4 mg discontinuation after 12 weeks of combination therapy. The switch to monotherapy with tadalafil 5 mg or tamsulosin 0.4 mg was associated with a worsening of LUTS and ED compared with the combination period. Total (+ 2.08 vs + 1.72, *p* = 0.38), voiding (+ 1.6 vs + 0.64, *p* = 0.45), and QoL (+ 0.64 vs + 0.52, *p* = 0.6) IPSS changes were comparable between the two groups. However, storage IPSS (+ 0.24 vs + 1.2, *p* = 0.04) and IIEF (− 1.6 vs − 4.4, *p* = 0.003) were significantly better in the tadalafil group, conversely, Qmax in the tamsulosin group (− 2.41 vs − 0.25, *p* = 0.001) [38••].

## Discussion

Tadalafil, in particular 5 mg/daily, proved to be effective for the treatment of both ED and LUTS due to BPH. Preclinical evidence demonstrated that penile tissue, along with bladder and prostate, is enriched of PDE5, thus rendering these tissues the preferential targets of PDE5 inhibitors and susceptible to their beneficial effects. Indeed, PDE5 inhibition is able not only to counteract BPH-associated intraprostatic inflammation but also to increase blood supply and tissues oxygenation, thus playing a crucial role in relieving LUTS. These mechanisms are also directly linked with the improvement of ED. Moreover, the proven action on smooth muscle cell tone strengthen, from a physiological point of view, the beneficial effect of tadalafil on LUTS. Therefore, the use of daily tadalafil 5 mg in monotherapy for the treatment of patients complaining both LUTS and ED appears more and more persuasive, thanks also to the good safety and tolerability profile, as shown in our systematic review. In fact, the incidence of TEAEs in the evaluated studies was similar to tamsulosin, without any negative impact on sexual function, in particular ejaculatory function, as reported by men treated with ABs [4–7, 33, 37••, 39].

However, severe LUTS, in particular linked to BOO, may not be satisfactorily relieved with tadalafil monotherapy, as also with ABs monotherapy. Whereby, several drug combinations, such as ABs and 5alpha reductase inhibitors (5ARIs), have been well established and are currently reported in the major urological guidelines as standard therapies. However, despite their efficacy in treating LUTS, their side effects, above all on sexual function, are mainly detrimental [2, 38••, 40–42].

In the last years, thanks to the good evidence, the association of tadalafil and tamsulosin has been approved too for the treatment of LUTS. Indeed, especially for younger patients, combination treatment represents a more tolerable treatment option for moderate to severe LUTS, above all when sexual function is already compromised. According to our reports, combination of daily tadalafil 5 mg and tamsulosin 0.4 mg showed an improvement of LUTS relief when compared to monotherapy with both the single drugs, retaining a good compliance and safety profile, despite a slight increase of TEAEs. Moreover, this combination may also be easily adapted to patients’ needs. In fact, discontinuation of PDE5-Is or ABs are easier and more modulable when compared to 5ARIs or antimuscarinics. Indeed, tadalafil or tamsulosin is rapidly active and can be easily and effectively reassumed [2, 28, 35, 37••].

## Conclusions

In conclusion, daily tadalafil, in particular at dosage of 5 mg, is effective for the treatments of LUTS/BPH and ED. All the available evidence shows that the occurrence of TEAEs is low and most of patients are “satisfied” by this treatment. Combination therapy of tadalafil 5 mg and tamsulosin 0.4 mg allows a further improvement of urinary symptoms and ED, against a higher rate of TAEs. Discontinuation of tamsulosin or tadalafil, after combination therapy, seems to allow a preservation of the results obtained for LUTS relief. However, tadalafil only is able to retain ED improvement.
